# Energy-Efficient Channel Coding Strategy for Underwater Acoustic Networks

**DOI:** 10.3390/s17040728

**Published:** 2017-03-31

**Authors:** Grasielli Barreto, Daniel H. Simão, Marcelo E. Pellenz, Richard D. Souza, Edgard Jamhour, Manoel C. Penna, Glauber Brante, Bruno S. Chang

**Affiliations:** 1 Postgraduate Program in Computer Science (PPGIa), Pontifical Catholic University of Parana, Paraná 80215-901, Curitiba, Brazil; grasibarreto@gmail.com (G.B.); ejamhour@gmail.com (E.J.); penna@ppgia.pucpr.br (M.C.P.); 2 Graduate Program in Electrical and Computer Engineering (CPGEI), Federal University of Technology, Paraná 80230-901, Curitiba, Brazil; hayashidadaniel@gmail.com (D.H.S.); gbrante@utfpr.edu.br (G.B.); bschang@utfpr.edu.br (B.S.C.); 3 Department of Electrical Engineering, Federal University of Santa Catarina, Santa Catarina 88040-900, Florianópolis, Brazil; richard.demo@ufsc.br

**Keywords:** underwater acoustic networks, forward error correction codes, fountain codes, energy efficiency

## Abstract

Underwater acoustic networks (UAN) allow for efficiently exploiting and monitoring the sub-aquatic environment. These networks are characterized by long propagation delays, error-prone channels and half-duplex communication. In this paper, we address the problem of energy-efficient communication through the use of optimized channel coding parameters. We consider a two-layer encoding scheme employing forward error correction (FEC) codes and fountain codes (FC) for UAN scenarios without feedback channels. We model and evaluate the energy consumption of different channel coding schemes for a *K*-distributed multipath channel. The parameters of the FEC encoding layer are optimized by selecting the optimal error correction capability and the code block size. The results show the best parameter choice as a function of the link distance and received signal-to-noise ratio.

## 1. Introduction

Underwater acoustic networks (UANs) are used in a wide range of applications [1], including biological ecosystem monitoring, oceanography data collection, fishing exploration, pollution control, sub-aquatic surveillance [2,3,4] and underwater autonomous vehicles [5]. Basically, a UAN consists of sensor nodes disposed at different depths in the underwater environment, which are interconnected by gateways using acoustic links and surface stations to receive and process the collected data. The underwater acoustic channel is affected by attenuation, noise and multipath propagation [6], which leads to long propagation delays, transmit errors and reduced channel bandwidth [7].

In order to tackle these problems, the UAN may employ channel coding techniques similar to those used in wireless sensor networks (WSNs); these techniques can improve the system performance and reduce the energy consumption. Energy efficiency in UAN may be even more important than in WSN because the impairments imposed by the underwater environment can be extremely challenging. Typically, in UAN communication, the transmit energy consumption is 100 times greater than the reception one [3]. Additionally, it may be difficult and costly to replace the batteries of the sensor nodes. Error control strategies, such as forward error correction (FEC) codes and fountain codes can be used in order to improve the transmit efficiency and reduce energy consumption at the transmitter and receiver. The use of fountain codes is motivated by the long propagation delays introduced by classical automatic repeat request (ARQ) schemes in the underwater acoustic channel, which may considerably reduce the throughput when multiple retransmissions are required. Moreover, ARQ schemes cannot be applied in scenarios when a feedback channel is not available. Additionally, the fountain code approach may be useful in multicast scenarios where receivers experience different channel conditions that are unknown to the transmitter.

Motivated by the proposed energy-efficient optimization of FEC coding schemes for WSNs presented in [8,9] and the benefits of fountain codes [10] for erasure channels, we investigate the energy efficiency by jointly using FEC and fountain coding schemes in underwater acoustic links. We aim at defining the best FEC encoding parameters for the mixed FEC/Fountain strategy in order to reduce the energy consumption in the communication link and therefore extend the lifetime of the UAN.

This paper proposes a mixed FEC/Fountain transmission strategy for the underwater acoustic channel. An energy-efficient communication link is obtained through the use of optimized FEC code parameters. We model and evaluate the energy consumption of different channel coding schemes for a *K*-distributed multipath fading channel. We determine the optimal coding parameters to be selected as a function of the link distance or operating signal-to-noise ratio (SNR) at the receiver. The results show that the energy consumption can be significantly reduced by the proposed optimization.

The remainder of this paper is organized as follows. Section 2 discusses related work. The underwater acoustic channel and the system model are described in Section 3. The proposed mixed FEC/Fountain transmit strategy is presented in Section 4. The results are shown in Section 5. Conclusions are summarized in Section 6.

## 2. Related Work

The use of optimized FEC channel coding strategies for energy saving has been extensively investigated for terrestrial wireless networks, as, for instance, in [8,9,11]. For instance, in [11], the authors investigate the trade-off between transmission and processing energy consumption in wireless sensor networks. The study identifies and selects the appropriate complexity of the error control code to be used in each sensor node, in order to maximize the network lifetime. A detailed study developed in [8] established the optimal coding rate selection as a function of the transmit distance between communicating nodes for Additive White Gaussian Noise (AWGN) and Rayleigh fading channels in terrestrial setups. The energy efficiency achievable by ARQ schemes when the code rate of the error-correcting code is optimized is investigated in [9].

In the case of UAN, the optimization of channel coding strategies has also been investigated due to their potential energy savings. In [12], we investigated the use of binary frequency-shift-keying (FSK) modulation and convolutional error correcting codes in UAN. The operation frequency, SNR and code rate are optimized for a target frame error rate (FER). The results show that the proper choice of code rate has a great impact on the overall energy consumption and lifetime of the underwater communication devices. In [13], it was analyzed how much energy is required to successfully transfer an information bit over a multi-hop underwater acoustic network while considering the optimum number of hops, retransmissions, code rate, and SNR. In [14], an optimization process is used to obtain the optimal code rate, SNR and modulation order for energy efficient underwater communications with ARQ.

The use of fountain codes in UAN was experimentally investigated in [15]. The transmitter sends coded packets with a predetermined redundancy over a simplex link with no feedback. The strategy uses Orthogonal Frequency Division Multiplexing (OFDM) and the authors investigate the relation between FER and the number of required transmissions. In [16], the use of random linear fountain codes associated with an adaptive power control strategy for energy savings in UANs is considered, in order to reduce the energy consumption. A recent study presented in [5] investigates a reliable transmit strategy for UAC which employs fountain codes and ARQ schemes. The authors optimize the number of transmitted coded packets in order to satisfy a pre-defined reliability at the receiver, assuming a stop-and-wait ARQ technique. An initial study of the energy consumption of UAN employing fountain codes is carried out in [17], whose main idea is to transmit an undetermined amount of coded bits so as to ensure a given target FER at the receiver without the need for retransmissions. Note that [17] employs a fixed number of coded bits, while the optimization in terms of the code rate may thus lead to a very small number of information bits, which may be a problem in longer distances, since the code rate becomes very small. In [18], a scheme in which the number of required coded packets is determined as to achieve a specified reliability criterion is proposed. The transmitter adjusts its power and the number of coded packets using the channel state information that is obtained via a feedback channel from the receiver.

A new protocol for underwater acoustic communication using fountain codes was proposed in [19]. However, the protocol employs a hybrid strategy using automatic repeat request (ARQ), which requires a feedback communication channel. In [20] the authors addressed the problem of stopping sets occurring during fountain decoding in the UAN communication. In particular, they focused on improving the fountain encoding and decoding algorithms.

A hybrid protocol, FOCAR, was proposed in [21]. It integrates fountain codes with hop-by-hop retransmission-upon-failure, requiring a feedback channel and employing selective repeat ARQ. They optimize the data block size on every intermediate node by using a centralized optimization procedure that requires packet error rate information from every hop. The study does not consider the use of specific FEC codes and their interaction with fountain codes.

In [22], the authors proposed a broadcasting algorithm for UANs, where a source node disseminates information to a number of nodes randomly placed within a given geographical area. A mathematical model to characterize the performance of a hybrid ARQ based fountain code scheme was derived. No practical broadcasting protocol was proposed and the use of FEC codes is not addressed.

In [23], a hybrid ARQ scheme based on fountain codes for the transmission of multicast messages in underwater channels is proposed. The scenario considers the use of fountain codes with a stop-and-wait ARQ strategy in order to enhance the performance of broadcast communications. The use and selection of FEC code parameters are not investigated.

In [24], a fountain-based reliable unicast transmission scheme by exploiting the estimating process of channel state information (CSI) is proposed. The scheme employs a specific class of fountain codes known as Raptor codes. In order to improve the goodput, the authors apply stochastic optimization to adapt the parity ratio. Our study differs from [24] in the sense that we consider a two-layered coding scheme and formulate the optimization process focusing on energy consumption. Additionally, we employ a relatively simple class of FEC codes (BCH codes), with respect to the implementation, which is a desirable characteristic for the encoding and decoding processes in sensor nodes with limited energy resources.

In [25], the authors investigate a layered coding approach that uses error-correction coding within each packet and erasure-correction coding across the packets. They addressed a wireless link scenario and determined the optimum trade-off in splitting redundancy between error-correction and erasure-correction codes. The results determine the optimum trade-off in splitting redundancy between error-correction coding and erasure-correction codes, which depends on the fading statistics and the average SNR of the wireless channel. The study assumes a theoretical premise that the system employs capacity-achieving channel coding. Their optimization aims at achieving a given error performance with a maximum rate. Differently, besides considering an underwater channel model and other related parameters, our approach considers practical block codes, fast fading and we model the optimization problem from an energy consumption perspective. Thus, in our work, the coding parameters are optimized focusing on maximizing the network energy resources, not the network rate, which may be more desirable in the case of sensor networks.

Against the above background, in this work, we propose a mixed FEC/fountain coding scheme for UAN using a two-layer approach, in which we are able to adjust the rate of the FEC code and the number of coded packets in the fountain code as to achieve a given target reliability while minimizing the energy consumption. Moreover, the proposed method operates without a feedback channel, being therefore suitable for multicast networks. Our study finds applicability in protocol development, network topology planning and network performance optimization.

## 3. Channel and System Model

In this section, we model the underwater acoustic channel in terms of path loss, additive noise and fading. Moreover, we also discuss the bit error probability (BEP) of binary non-coherent frequency shift keying modulation and the relationship between acoustic and electric transmit power.

The path loss experienced in an underwater acoustic channel can be written as [26]

(1) $$A\left(l,f\right)={\left(1000\cdot l\right)}^{k}a{\left(f\right)}^{l},$$

where *l* is the transmit distance in km, *f* is the frequency in kHz, *k* is the spreading factor and a(f) is the absorption coefficient. The spreading factor describes the geometry of the propagation. We assume a practical spreading factor of k=1.5. The absorption coefficient is calculated according to the Thorp’s expression as [27]

(2) $$10\log_{10}\left[a(f)\right]=\frac{0.11f^{2}}{1+f^{2}}+\frac{44f^{2}}{4100+f^{2}}+\frac{2.75f^{2}}{10^{4}}+0.003\;\;\mathrm{dB}/\mathrm{km}.$$

The additive noise in the acoustic underwater channel is modeled considering four basic sources: thermal noise $N_{th}\left(f\right)$, waves $N_{w}\left(f\right)$, shipping $N_{s}\left(f\right)$ and turbulence $N_{t}\left(f\right)$. The following empirical formulas define these noise components, in dBreμPa per Hz [6]:

(3) $$10\log_{10}N_{th}\left(f\right)=-15+20\log_{10}\left(f\right),$$

(4) $$\begin{array}{c} \begin{array}{c} 10\log_{10}N_{w}\left(f\right)=50+7.5\sqrt{w}+20\log_{10}\left(f\right)-40\log_{10}\left(f+0.4\right), \end{array} \end{array}$$

(5) $$\begin{array}{c} \begin{array}{c} 10\log_{10}N_{s}\left(f\right)=40+20\left(s-0.5\right)+26\log\left(f\right)-60\log_{10}\left(f+0.03\right), \end{array} \end{array}$$

(6) $$10\log_{10}N_{t}\left(f\right)=17-30\log_{10}\left(f\right).$$

In $N_{w}\left(f\right),$ the wind speed *w* is given in m/s. The shipping activity parameter *s* in $N_{s}\left(f\right)$ has a range from 0 to 1, from low to high activity, respectively. The overall noise power spectral density is thus

(7) $$N\left(f\right)=N_{th}\left(f\right)+N_{w}\left(f\right)+N_{s}\left(f\right)+N_{t}\left(f\right)\;\;\;\mathrm{Pa}/\mathrm{Hz}.$$

Given that the acoustic transmit and received power are, respectively, $P_{r}$ and $P_{t}$, the average received signal-to-noise ratio (SNR) is

(8) $$\bar{\gamma }\left(l,f\right)=\frac{P_{r}}{N(f)B(f)}=\frac{P_{t}/A\left(l,f\right)}{N(f)B(f)}\propto \frac{1}{A(l,f)N(f)},$$

where B(f) is the usable signal bandwidth around the center frequency. We assume a narrow-band model, where the channel gain can be approximated as being constant in the signal bandwidth [28]. The A(l,f)N(f) product shows the frequency-dependent part of the SNR. Therefore, for a specific transmit distance *l*, there exists an optimal carrier frequency that maximizes the SNR.

According to experimental results, it was shown in [29] that the small-scale propagation effects, due to multipath fading, in the underwater acoustic channel can be well modeled by the *K*-distribution probability density function,

(9) $$f\left(x\right)=\frac{4}{\sqrt{\alpha }\;\Gamma \left(\nu \right)}{\left(\frac{x}{\sqrt{\alpha }}\right)}^{\nu }K_{\nu -1}\left(\frac{2x}{\sqrt{\alpha }}\right),$$

where ν is a shape parameter, α is a scale parameter, $K_{\nu -1}\left(\cdot \right)$ is the modified Bessel function of second kind and order ν−1, and Γ(·) is the Gamma function. We assume a scenario where the transmission system operates at a fixed frequency and under a fast fading channel [30]. Moreover, in our subsequent analysis, we consider a non-coherent binary FSK modulation scheme, whose BEP in the *K*-distributed fading is given by [29]

(10) $$P_{b}\left(\bar{\gamma }\right)=\frac{\nu }{\Gamma (\nu )}\;\int_{0}^{\infty }\frac{u^{\left(\nu -1\right)}e^{-u}}{2\nu +u\;\bar{\gamma }}\;du,$$

where the average received SNR is expressed as $\bar{\gamma }=E_{b}/N\left(f\right)$. The parameter $E_{b}=P_{r}/R_{b}$ represents the average energy per bit.

The electric transmit power $P_{PA}$ of the power amplifier (PA) can be obtained from the acoustic transmit power $P_{t}$ (in μPa) using the following relation [31]

(11) $$P_{PA}=\left(P_{t}\cdot 10^{-17.2}\right)/\varphi \;\;\left(\mathrm{Watts}\right),$$

where $10^{-17.2}$ is a conversion factor and φ is the overall efficiency of the electric power amplifier plus transducer. In practice, a minimum transmit power level is always required [31], independent of the link distance *l*. The total electric power for a single transmit attempt over the acoustic link can be written as

(12) $$P_{total}=P_{tx}^{el}+\max\left(P_{PA},P_{PA}^{min}\right)+P_{rx}^{el}\;\;\left(\mathrm{Watts}\right),$$

where $P_{PA}^{min}$ is the minimum transmit power, $P_{tx}^{el}$ and $P_{rx}^{el}$ are the electric power consumptions (in Watts) of the transmit and receive circuits, respectively.

## 4. Proposed Transmit Strategy

In this section, we present the proposed mixed coding strategy, which jointly employs FEC and fountain codes in UAN. Our goal is to optimize the coding parameters as a function of the SNR and the transmit distance between nodes, in order to minimize the energy consumption in the communication link for a given target error probability.

**Figure 1:**
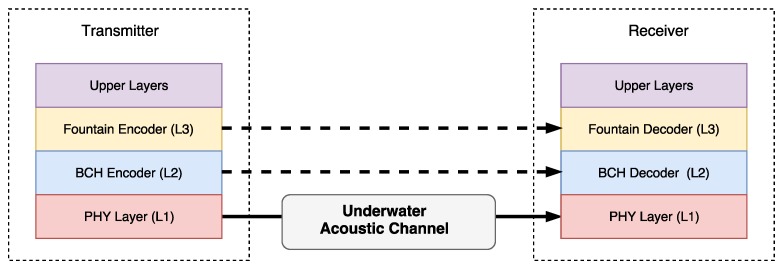
System model.

The proposed scheme is shown in Figure 1 and employs two encoding layers over the physical layer (L1). The fountain code layer (L3) enables reliable transmission without the need of a feedback channel. The FEC encoder layer (L2) gives support to L3 by reducing the FER and improving the FC performance.

At the transmitter, the L2 layer needs to wait for a group of information bits in order to generate a coded data block. In this paper, we consider BCH block codes of rate r=k/n at the FEC encoder layer because these codes have already been used in underwater communication systems [32]. However, this study can be extended for other types of FEC codes as well. Moreover, the BCH codewords are encapsulated into the payload of the L1 layer. We assume that a single packet at the output of the random linear coding (RLC) encoder layer has size *p* bits, where p>k. Depending on the BCH encoding parameters, an RLC coded packet (the output of the encoding procedure in Equation (14)) needs $N_{p}=p/k$ FEC packets to be encapsulated into the FEC code blocks.

The fountain code layer considers transmission rounds with a group of *M* coded packets coming from the upper layers. These packets are encoded into N≥M packets and sent over the acoustic link. In particular, in the fountain code layer, we assume the use of RLC where the *M* data packets of size *p* bits are each stored at the transmitter buffer as rows of a matrix P, where

(13) $$P=\left[\begin{array}{cccc} m_{11} & m_{12} & \cdots & m_{1p}\\ m_{21} & m_{22} & \cdots & m_{2p}\\ \vdots & \vdots & \ddots & \vdots \\ m_{M1} & m_{M2} & \cdots & m_{Mp} \end{array}\right].$$

**Figure 2:**
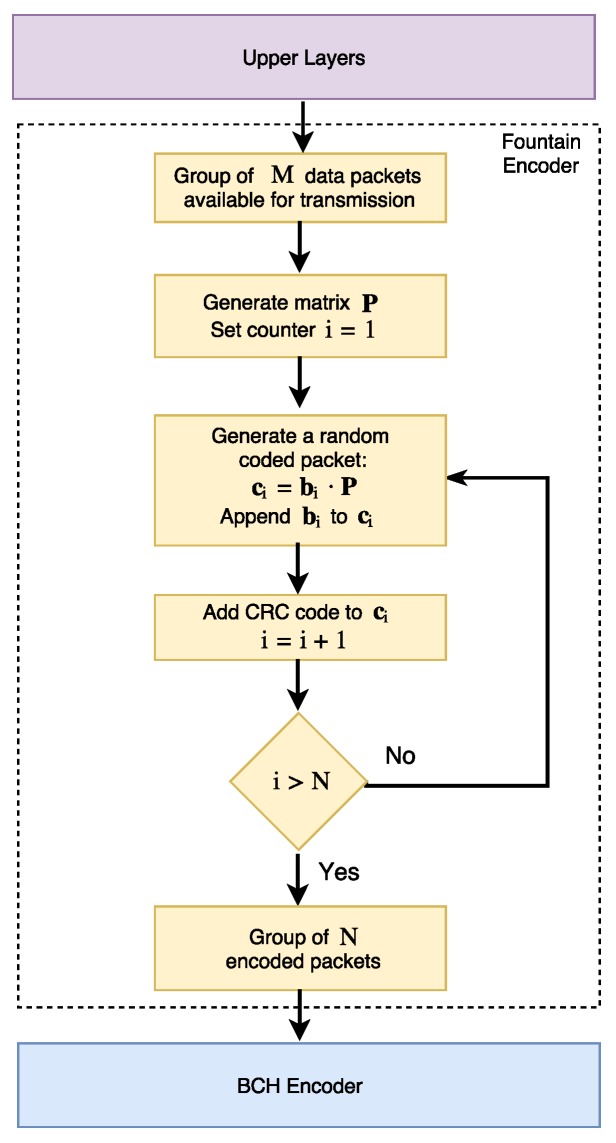
Fountain encoder structure (adapted from [15]).

A coded packet $c_{i}$ is generated as

(14) $$c_{i}=b_{i}\cdot P,$$

where $b_{i}=\left[b_{1},b_{2},\ldots ,b_{M}\right]$ contains the random coefficients used to encode the *i*-th data packet. We assume that operations are performed over the Galois field GF(2). The group of *N* encoded packets at L3 are sent to the FEC encoding layer. A detailed description of the encoding structure [15] is shown in Figure 2.

**Figure 3:**
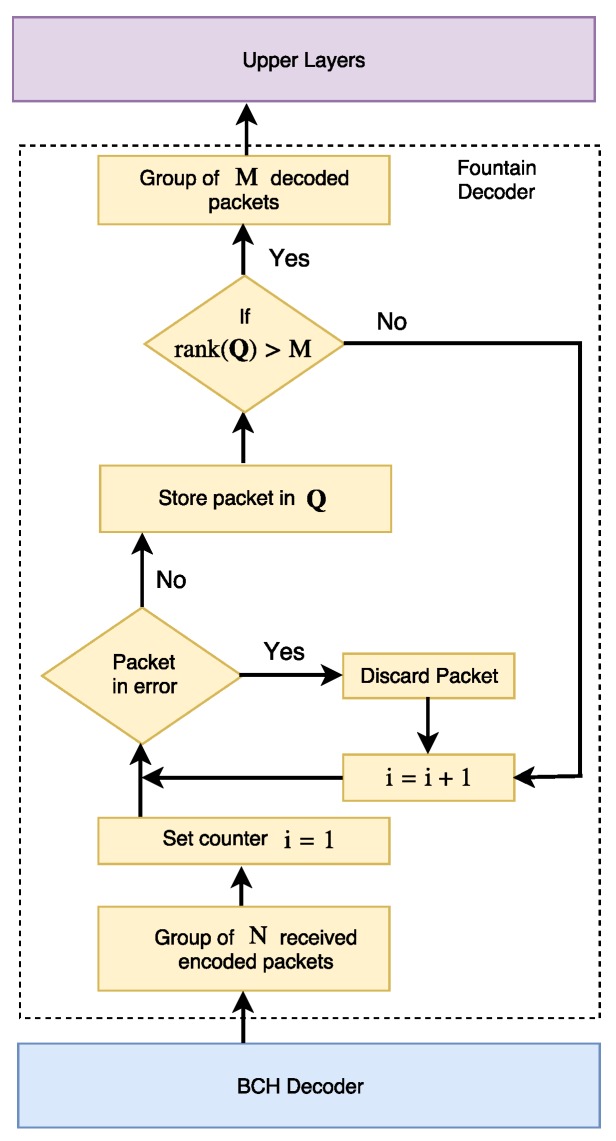
Fountain decoder structure (adapted from [15]).

At the receiver, each packet is checked for errors at the L3 layer. If a packet does not pass a standard procedure such as cyclic redundancy check (CRC), then it is discarded. The valid received packets are stored in a matrix Q and the random coding coefficients are removed from the packet and stored in a matrix A. When rank(Q)=M it means that there are *M* linearly independent combinations of the original packets that can be decoded as

(15) $$P=A^{-1}\cdot Q,$$

where $A^{-1}$ denotes the inverse of matrix A. The decoder structure [15] is shown in Figure 3. The fountain decoder operates with a packet success delivery probability, $P_{s}$, which is given by [16]

(16) Ps=∑m=MNNm1−PfmPf(N−m),

where $P_{f}$ is the FER perceived at the L3 layer. The required number of encoded packets, *N*, to achieve a target packet success delivery probability $P_{s}^{*}<1$ at the receiver depends on $P_{f}$.

Considering the L2 layer operation at the receiver, for a given BCH code with rate r=k/n, the FER perceived by the FEC decoder is given by

(17) PfBCH=1−∑j=0tnjPb(γ¯)j1−Pb(γ¯)n−j,

where *t* is the code error correction capability, *n* is the codeword length and $P_{b}\left(\bar{\gamma }\right)$ is the bit error probability given by Equation (10). In this work, we consider the ensemble of BCH codes presented in Table 1 [33]. Depending on the application requirements in terms of frame size, different BCH codes may be used. Let us recall that the a single packet of *p* bits at the output of the L3 encoder is encapsulated in $N_{p}=\lceil p/k\rceil$ BCH codewords, where ⌈x⌉ denotes the nearest integer greater than or equal to *x*. The probablity that such a L3 packet is wrongly decoded at L2 decoding of the receiver is

(18) $$P_{f}^{PHY}=1-{\left(1-P_{f}^{BCH}\right)}^{N_{p}},$$

where $P_{f}^{BCH}$ is defined according to Equation (17). Therefore, at the receiver side, the packet success delivery probability at the L3 layer perceives a FER given by $P_{f}=P_{f}^{PHY}$, which can be plugged into Equation (16) to compute the error probability at the L3 decoding. The transmit energy consumption necessary to recover the *M* information-bearing packets is

(19) $$E=\left(\frac{1}{r}\right)\frac{P_{t}^{el}}{R_{b}}\left(\frac{N}{M}\right).$$

The above equation is composed of a product of three terms. The first one relates to the redundancy introduced by the BCH code. The second term represents the energy consumption per transmitted bit, $E_{b}$. The last one refers to the additional energy consumption imposed by the fountain encoder. It is important to point out that the energy consumption defined by Equation (19) is a simplified energy model. Just for simplification, without affecting the relative results, we assume a negligible baseband processing consumption. A precise model for terrestrial wireless sensor networks can be found in [8], which includes the start-up, baseband processing and reception energy consumption. Our goal is to minimize the energy consumption given by Equation (19), formally defined as

(20) $$\begin{array}{cc} \underset{r=k/n}{\mathrm{minimize}} & E,\\ \mathrm{subject}\;\mathrm{to} & P_{s}^{*}<1,\;M,\;p, \end{array}$$

where $P_{s}^{*}$ denotes the target packet success delivery probability. The energy consumption can be optimized in terms of the required received SNR or communication link distance.

## 5. Results

Our experimental setup uses the previously described mathematical framework to analyze the system performance as a function of several parameters. Moreover, the employed system parameters are described in Table 1 and Table 2, which follow typical implementations for underwater acoustic sensors. The proposed transmit strategy was numerically evaluated in terms of the FEC coding rate at L2, for given *M* and *p*, which minimizes the energy consumption for a given target success delivery probability, as a function of either the received signal-to-noise ratio or the transmit link distance. We consider an acoustic link whose system parameters are listed in Table 2, while some of these parameters are taken from the WHOI modem [30,34]. In this section, all analysis in terms of SNR refers to the average SNR.

### 5.1. Signal-to-Noise Ratio Analysis

**Figure 4:**
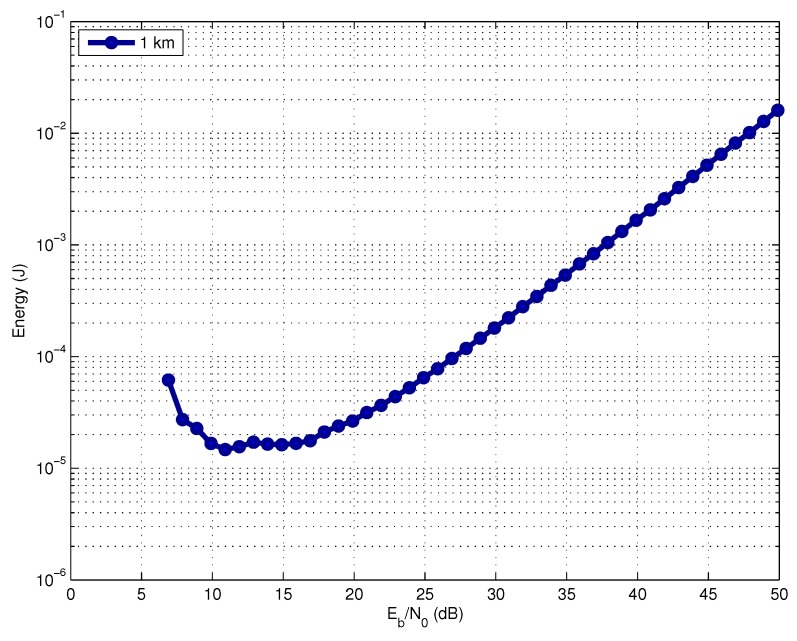
Energy consumption versus operating SNR.

In Figure 4, we analyze the energy consumption as a function of the operating SNR for a link distance of 1 km, assuming a target success delivery probability $P_{s}^{*}=0.999$. This result considers optimized BCH codes for every SNR value.

We can observe that the most efficiently operating SNR range in terms of energy consumption is between 10 dB and 15 dB for all link distances. For SNR values smaller than 10 dB, the energy consumption increases. This occurs because the FER increases and consequently the number of code packet transmissions required by the fountain code to correctly deliver the *M* information-bearing packets. On the other hand, for SNR values above 15 dB, it is necessary to excessively increase the transmit power, which also leads to an increasing energy consumption. In practice, however, an acoustic modem imposes limits to the maximum transmit power. Therefore, depending on the link distance, we cannot always achieve the optimal required SNR at the receiver.

**Figure 5:**
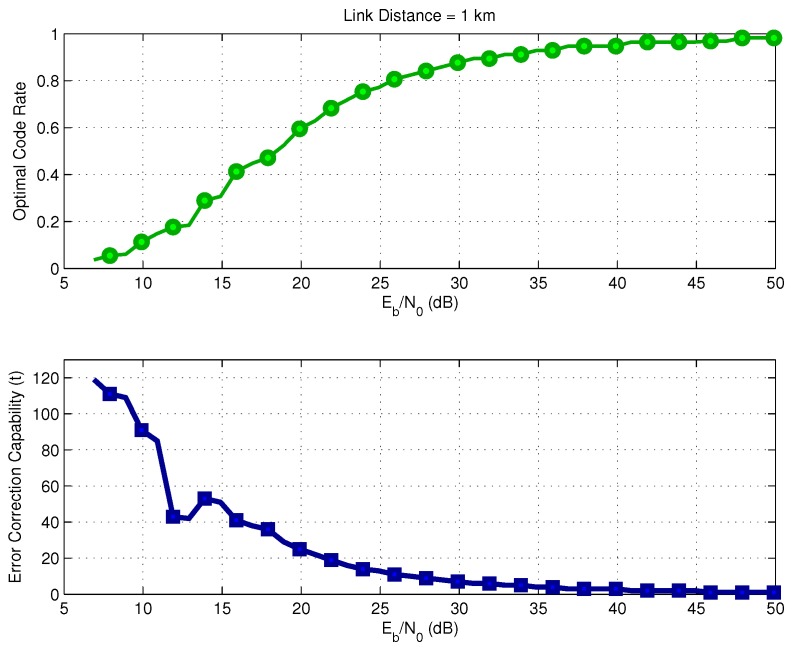
Optimal BCH code rate versus SNR for $P_{s}^{*}=0.999$.

Figure 5 shows the optimal FEC code rate r=k/n and the error correction capability *t*, as a function of the SNR for a frame size p=128 bytes and link distance of 1 km. The target success delivery probability is $P_{s}^{*}=0.999$. When the receiver operates with a higher SNR, the optimal rate approaches one, which indicates the need for lower redundancy because the link probability of error is very small. A similar behavior in terms of optimal code rate selection was observed for other link distances, whose results were omitted for sake of brevity.

**Figure 6:**
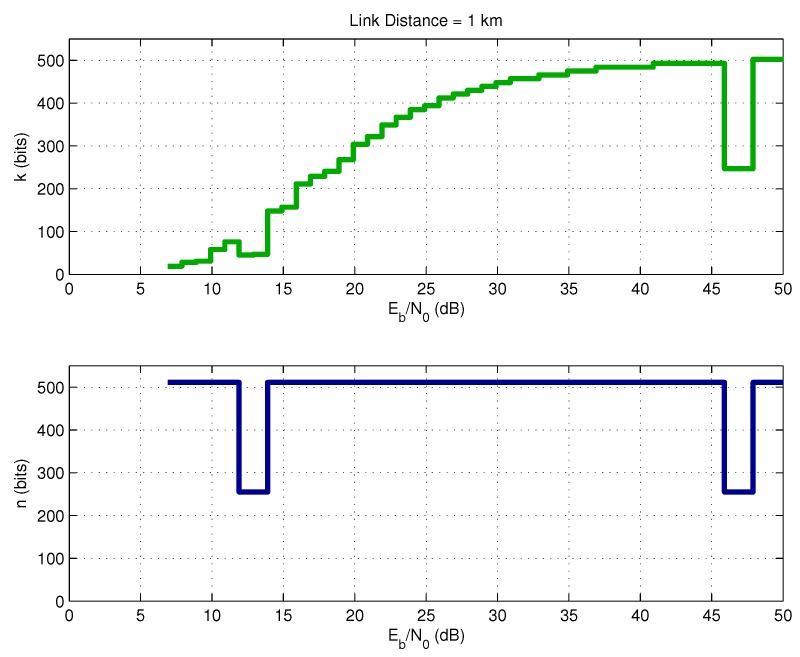
Optimal BCH parameters.

Figure 6 shows the exact selection of parameters *k* and *n* associated with the optimal coding rate *r* shown in Figure 5. It is important to point out that, in the intervals 10–15 dB and 45–50 dB, the selected code rate was $r_{1}=k/n=247/255$ instead of $r_{2}=502/511$. The reason is that $r_{1}$ is a little smaller than $r_{2}$, which causes a small reduction in terms of energy consumption.

**Figure 7:**
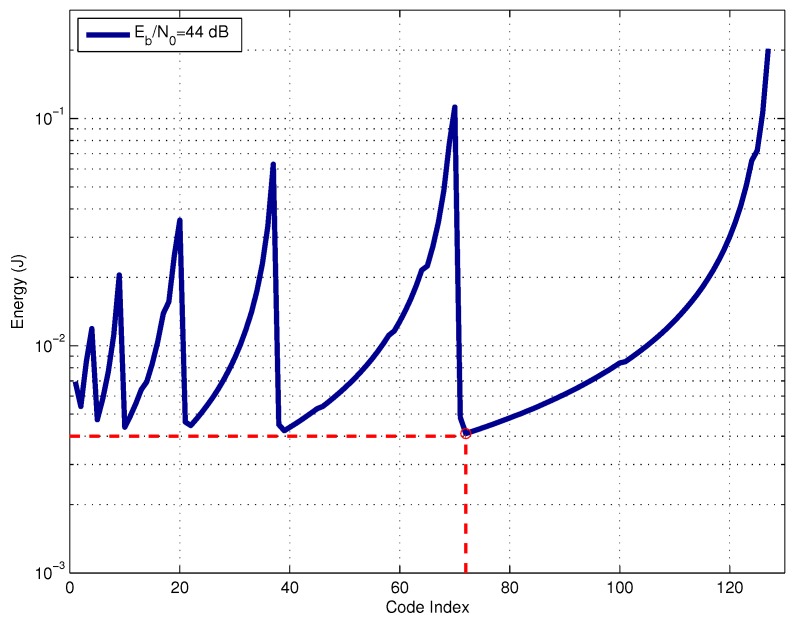
Energy consumption for different codes at a fixed SNR.

Figure 7 shows the energy consumption for all FEC codes in Table 1, assuming a specific SNR of $E_{b}/N_{0}=44\;\mathrm{dB}$. The *x*-axis represents code index, starting from code (n,k,t)=(7,4,1) (code index 1) up to (n,k,t)=(511,10,121) (code index 127). The main idea is to show the energy consumption level generated by each code if it is used in the transmission system. The operation SNR value of $E_{b}/N_{0}=44\;\mathrm{dB}$ was chosen by didactic methods. At this SNR level, all BCH codes are feasible choices, leading to reasonable values of energy consumption. The same analysis could be done for lower SNR values; however, in this case, not all of the codes of Table 1 could be used because the energy consumption is very high for some of them due to the number of required transmissions at the L3 layer. The red dashed line indicates the optimal code selection for operation at $E_{b}/N_{0}=44\;\mathrm{dB}$.

### 5.2. Link Distance Analysis

**Figure 8:**
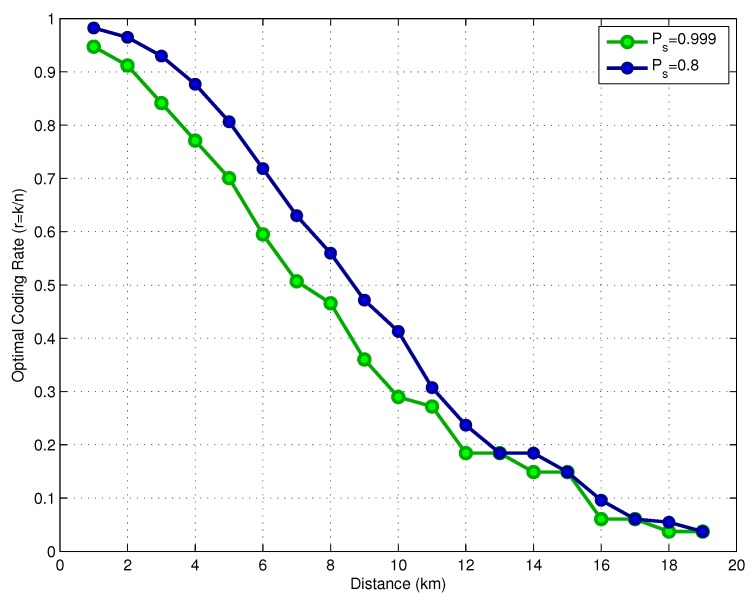
Optimal coding rate versus distance.

**Figure 9:**
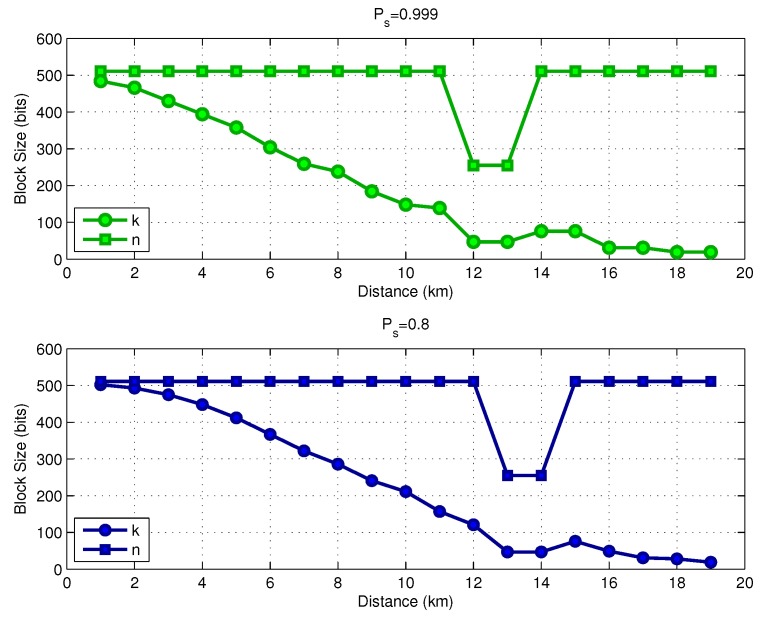
Energy consumption versus operating SNR (p=1024 bits).

**Figure 10:**
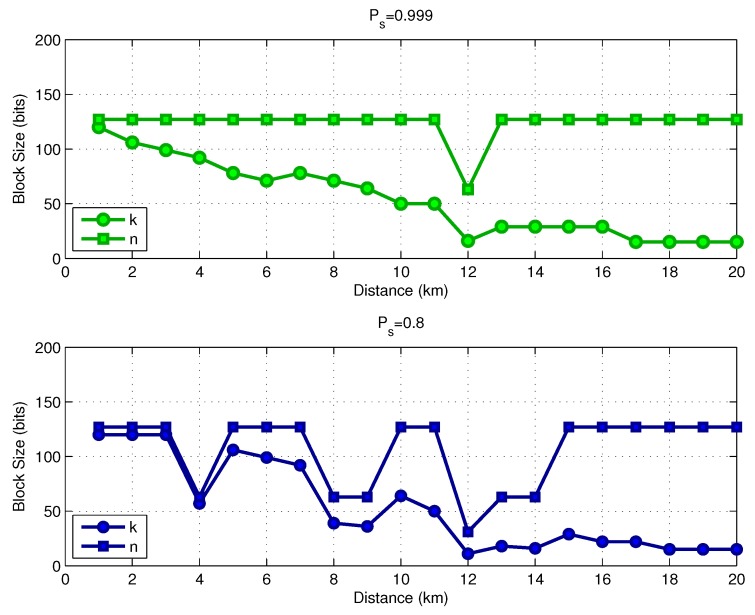
Energy consumption versus operating SNR (p=128 bits).

In Figure 8, we show the optimal code rate as a function of the link distance, assuming a transmit power $P_{t}=160\;\mathrm{dB}\;\mathrm{re}\;\mu \mathrm{Pa}$. The results consider two values for the target success delivery probability, $P_{s}^{*}=0.999$ and $P_{s}^{*}=0.8$. The required coding rate reduces with the distance, which means that we require a more powerful code for larger distances as the transmit power is limited. If the system is allowed to operate with a lower target success delivery probability ($P_{s}^{*}=0.8$), the link can use a higher coding rate, saving energy. Figure 9 shows the optimal parameters *k* and *n* for both values of $P_{s}^{*}$. Observe that in the distance range between 12 and 14 km, the selected codeword size was n=255 instead of n=511, as previously discussed. Figure 10 shows the optimal FEC parameters for a reduced block size of p=128 bits. In essence, the general conclusions are the same as for the case of p=1024 bits, with the main difference that, when considering p=128, the optimal choices of *k* and *n* are much more diverse than from those in the case of p=1024.

**Figure 11:**
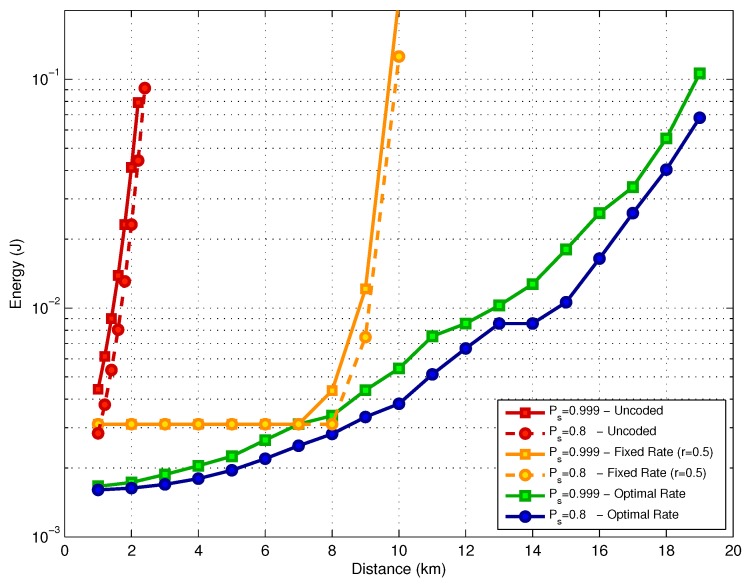
Energy consumption versus distance.

In Figure 11, we present a comparison of three different schemes in terms of energy consumption. The first one uses an uncoded L2 layer, while the second one uses a fixed code rate at the L2 layer (r=1/2); finally, the third one is the proposed mixed scheme with an optimized FEC coding rate. Note that the three schemes use the same fountain code.

For the uncoded scheme at the L2 layer, the energy consumption increases 30 times when the link distance goes from 1 km to 2 km, for both values of $P_{s}^{*}$. In the second scheme, when a fixed code rate (r=1/2) is employed, we can observe two distinct performance phases. From 1 km to 8 km, the energy consumption is kept constant because the channel code is able to compensate for the SNR degradation which occurs when the distance increases. The energy consumption at this point is above the consumption that would be achieved with an optimized scheme because the choice of the fixed rate r=1/2 is not optimal. For link distances beyond 8 km, the channel degradation is too severe, which results in an increased number of coded packets transmitted at the L3 layer. This results in a fast increase in the energy consumption. The third scheme uses the proposed mixed coding strategy with optimized code rate. In such a case, we achieve the best performance among the three options for the two target success delivery probabilities under analysis.

Based on the numerical results, we can determine the parameters (n,k,t) that must be chosen for a particular communication link. These parameters can be determined either from the average signal-to-noise ratio (SNR) or the link distance. From the practical point of view, the choice of BCH parameters can be performed in the planning phase or in the operating phase of the network. In the UAN planning phase, the parameters can be chosen based on the link distance between two nodes. During the network operation phase, the BCH code parameters can be negotiated between two nodes by using, for instance, a control protocol. The idea is to initially estimate the average received SNR and then select the respective optimized BCH code parameters. Considering the case where p=1024 bits (128 bytes), the parameters *n* and *k* are obtained from Figure 6 and Figure 9. The error correction capability, *t*, can be read from Table 1.

## 6. Conclusions

In this paper, we addressed the problem of energy-efficient communication in UAN through the use of an optimized mixed FEC/fountain channel coding scheme. Numerical results demonstrate the potential advantages of the proposed scheme in terms of energy consumption when compared to uncoded transmission and to the case of non-optimized (fixed) parameters. The proposed strategy leads to a graceful degradation in terms of energy efficiency with the increase of link distance, leading to an enlarged range for communication among nodes in UAN, while also increasing the network lifetime.

## Figures and Tables

**Table 1:** BCH codes.

*n*	*k*	*t*	*n*	*k*	*t*	*n*	*k*	*t*
**7**	4	1	**255**	163	12	**511**	268	29
**15**	11	1		155	13		259	30
	7	2		147	14		250	31
	5	3		139	15		241	36
**31**	26	1		131	18		238	37
	21	2		123	19		229	38
	16	3		115	21		220	39
	11	5		107	22		211	41
	6	7		99	23		202	42
**63**	57	1		91	25		193	43
	51	2		87	26		184	45
	45	3		79	27		175	46
	39	4		71	29		166	47
	36	5		63	30		157	51
	30	6		55	31		148	53
	24	7		47	42		139	54
	18	10		45	43		130	55
	16	11		37	45		121	58
	10	13		29	47		112	59
	7	15		21	55		103	61
**127**	120	1		13	59		94	62
	113	2		9	63		85	63
	106	3	**511**	502	1		76	85
	99	4		493	2		67	87
	92	5		484	3		58	91
	85	6		475	4		49	93
	78	7		466	5		40	95
	71	9		457	6		31	109
	64	10		448	7		28	111
	57	11		439	8		19	119
	50	13		430	9		10	121
	43	14		421	10			
	36	15		412	11			
	29	21		403	12			
	22	23		394	13			
	15	27		385	14			
	8	31		376	15			
**255**	247	1		367	16			
	239	2		358	18			
	231	3		349	19			
	223	4		340	20			
	215	5		331	21			
	207	6		322	22			
	199	7		313	23			
	191	8		304	25			
	187	9		295	26			
	179	10		286	27			
	171	11		277	28			

**Table 2:** System parameters.

Parameter	Description	Value
*k*	Spreading Factor	1.5
*s*	Shipping Activity	0.5
*w*	Wind Speed (m/s)	0
*B*	Channel Bandwidth (Hz) [30]	320
$R_{b}$	Bit Rate (bits/s) [30]	160
$P_{t}$	Transmit Power $\left(\mathrm{dB}\;\mathrm{re}\;{}^{-}\mathrm{Pa}\right)$	120–190
φ	Efficiency of the Power Amplifier (PA) + Transducer [31]	0.25
*p*	Frame Payload (bytes) [30]	128
*M*	Number of Data Packets per Fountain Coding Round [16]	10
$P_{s}$	Target Success Delivery Probability [16]	{0.999 , 0.8}
ν	*K*-distribution Shape Parameter [29]	1.5
α	*K*-distribution Scale Parameter [29]	$\nu^{-1}$
